# Influence of Training With Corrective Feedback Devices on Cardiopulmonary Resuscitation Skills Acquisition and Retention: Systematic Review and Meta-Analysis

**DOI:** 10.2196/59720

**Published:** 2024-12-19

**Authors:** Abel Nicolau, Inês Jorge, Pedro Vieira-Marques, Carla Sa-Couto

**Affiliations:** 1 RISE-Health, Faculty of Medicine, University of Porto Porto Portugal; 2 RISE-Health, Department of Community Medicine, Information and Health Decision Sciences, Faculty of Medicine, University of Porto Porto Portugal

**Keywords:** cardiopulmonary resuscitation, CPR quality, resuscitation training, corrective feedback devices, skills acquisition, skills retention, systematic review, evidence-based research, meta-analysis, feedback devices, PRISMA

## Abstract

**Background:**

Several studies related to the use of corrective feedback devices in cardiopulmonary resuscitation training, with different populations, training methodologies, and equipment, present distinct results regarding the influence of this technology.

**Objective:**

This systematic review and meta-analysis aimed to examine the impact of corrective feedback devices in cardiopulmonary resuscitation skills acquisition and retention for laypeople and health care professionals. Training duration was also studied.

**Methods:**

The search was conducted in PubMed, Web of Science, and Scopus from January 2015 to December 2023. Eligible randomized controlled trials compared technology-based training incorporating corrective feedback with standard training. Outcomes of interest were the quality of chest compression–related components. The risk of bias was assessed using the Cochrane tool. A meta-analysis was used to explore the heterogeneity of the selected studies.

**Results:**

In total, 20 studies were included. Overall, it was reported that corrective feedback devices used during training had a positive impact on both skills acquisition and retention. Medium to high heterogeneity was observed.

**Conclusions:**

This systematic review and meta-analysis suggest that corrective feedback devices enhance skills acquisition and retention over time. Considering the medium to high heterogeneity observed, these findings should be interpreted with caution. More standardized, high-quality studies are needed.

**Trial Registration:**

PROSPERO CRD42021240953; https://www.crd.york.ac.uk/prospero/display_record.php?RecordID=240953

## Introduction

Sudden cardiac arrest (SCA) is a global leading cause of death [1], resulting from the interruption of cardiac mechanical activity and confirmed by the absence of signs of circulation [2]. In 2021, the estimated incidence of out-of-hospital cardiac arrests (OHCAs) in the United States was 92.3 cases per 100,000 individuals, with an adult rate of survival to hospital discharge following OHCA of 9.1% [2]. In Europe, the most recent estimation of the incidence of OHCA was 89 cases per 100,000 individuals, with an average survival rate of 8% [1,3,4]. Regarding in-hospital cardiac arrest, based on the latest data from the United Kingdom and the United States, there are around 1.6 to 2.85 cases of in-hospital cardiac arrest per 1000 hospital admissions, with a survival rate from 18.4% to 25.6% [5]. Globally, the incidence of SCA keeps increasing, representing a significant public health problem [1].

Early and high-quality cardiopulmonary resuscitation (CPR) can double or triple the survival rate from SCA [6,7]. CPR is an emergency lifesaving intervention for SCA that can be provided by both laypeople and health care professionals. It consists of applying chest compressions and rescue breaths to the victim. For adults, a 30:2 compressions-to-breaths ratio is recommended [7,8]. Chest compression is the most important component of CPR, as it provides organ perfusion and oxygenation during SCA [7,8]. Chest compression–only approach is acceptable if lay rescuers are untrained or are reluctant to provide rescue breaths [7]. The American Heart Association and the European Resuscitation Council have established detailed guidelines [7,8] for performing high-quality chest compressions during CPR. Key recommendations include placing hands on the lower half of the sternum, ensuring a compression depth of at least 5 cm (2 inches) but not exceeding 6 cm (2.4 inches), maintaining a compression rate of 100 to 120 compressions per minute, with minimal interruptions, and allowing the chest wall to fully recoil after each compression. Additionally, if possible, chest compressions should be performed on a firm surface.

Improving CPR quality by adhering to established guidelines has been shown to positively impact patient outcomes, particularly by increasing the survival rate of SCA victims [9]. To achieve this, training is recommended for both laypeople and health care professionals. Such training aims to improve CPR skills, thereby elevating the quality of the maneuvers performed and increasing patient safety [10]. Studies have shown that the quality of CPR performed by health care professionals and laypeople is usually poor [11-14]. CPR training may improve providers’ confidence and performance [15-17]. A typical CPR training session is delivered at a single period of time, with no interruptions, and supervised by certified instructors [18].

During a CPR training session, the feedback provided to the participant is crucial; however, the instructor’s assessment of the chest compression quality often tends to be subjective and suboptimal [19,20]. The effectiveness of the training methods of CPR instructors could be significantly enhanced by incorporating supportive devices, which offer quantitative assessments and objective feedback on chest compression quality [14,19,21].

A CPR training device with corrective feedback enhances the learning experience by offering audiovisual feedback based on assessed CPR quality parameters. These devices typically provide real-time feedback, allowing the participant to immediately adjust and improve their technique while performing maneuvers [22-25]. Since 2010, both the American Heart Association and European Resuscitation Council guidelines have endorsed the use of prompts and feedback devices for CPR training, recognizing their role in promoting skills acquisition and retention [26,27]. Initially, these guidelines broadly categorized feedback devices, including those offering general guidance without correcting skills (eg, metronome). However, as of 2015, the guidelines clearly distinguished between devices that provide corrective feedback and those that offer general guidance [28].

In the literature, there are several studies related to the use of both corrective and guiding feedback devices in CPR training, involving different target populations (from laypeople to health care professionals), training methodologies (different durations, formats, and assessment methods), and equipment. This diversity in study design makes it difficult to generalize their results. Furthermore, given the impact of corrective feedback devices in CPR training and the high potential to contribute to better quality in CPR maneuvers, it is of most relevance to study their influence through evidence synthesis studies.

To our knowledge, the existing reviews on this topic [29-32] lack at least 1 key aspect: (1) a focus on corrective feedback devices, as recommended by the recent guidelines [18], excluding studies on guiding devices; (2) an expanded target audience that includes both laypeople and health care professionals, training on both adult and pediatric manikins; (3) the provision of a reliable meta-analysis; and (4) the exclusion of studies focusing on real emergency setting or adjacent aspects to CPR training (eg, testing feedback devices).

Some existing reviews addressing similar topics are based on outdated guidelines [29,30] or have a broader scope, including the analysis of feedback devices in real clinical settings [29-31]. The distinction between studies focusing on specific target groups (eg, laypeople [32] or health care professionals [30,31]), along with varying methodologies and the exclusion of meta-analysis [29,31], highlights the limitations of current research.

This systematic review and meta-analysis aim to comprehensively survey the literature to characterize and assess the impact of corrective feedback devices on CPR skills acquisition and retention for both laypeople and health care professionals compared to the absence of these feedback devices. Training duration was also studied.

## Methods

### Study Design

This study is a systematic review and meta-analysis following the PRISMA (Preferred Reporting Items for Systematic Reviews and Meta-Analyses) 2020 guidelines [33], as shown in Multimedia Appendix 1, and the Cochrane Handbook for Systematic Reviews of Interventions [34]. The study protocol was developed by the research team and registered in PROSPERO (registration CRD42021240953). The protocol can be accessed through the PROSPERO website. No ethics approval was required.

### Information Sources and Search Strategy

Searches were conducted in MEDLINE (through PubMed), Web of Science, and Scopus, including publications between January 2015 and December 2023. The search strategy and query were designed to include all relevant publications considering the established eligibility criteria. The search query is presented in Textbox 1 and Multimedia Appendix 2 and includes keywords based on three categories: (1) CPR, (2) feedback devices, and (3) outcomes. Each category considered several keywords that were searched in the publications’ titles and abstracts. The search strategy was adapted to all included databases. The reference lists of relevant systematic reviews were screened to identify additional studies. Duplicates were removed using Mendeley Reference Manager and, later, verified using CADIMA software (Julius Kühn-Institut) [35].

**General search strategy.**

Publications from January 2015 to December 2023 in MEDLINE, Web of Science, and Scopus

1. Cardiopulmonary resuscitation [tab]

2. CPR [tab]

3. Basic life support [tab]

4. BLS [tab]

5. Resuscitation [tab]

6. 1 OR 2 OR 3 OR 4 OR 5

7. Training [tab]

8. Feedback [tab]

9. Manikin* [tab]

10. Device* [tab]

11. Prompt* [tab]

12. Audiovisual [tab]

13. Technology [tab]

14. Simulat* [tab]

15. 7 OR 8 OR 9 OR 10 OR 11 OR 12 OR 13 OR 14

16. Quality [tab]

17. Performance [tab]

18. Compression* [tab]

19. Acquisition [tab]

20. Retention [tab]

21. 16 OR 17 OR 18 OR 19 OR 20

22. 6 AND 15 AND 21

### Eligibility Criteria

The PICOS (participants, intervention, control, outcomes, and studies) framework was used to support the identification of the eligibility criteria.

- The participants or population (P) was either laypeople or health care professionals. No age limitations were included.
- The intervention (I) was the use of CPR training devices with feedback, providing corrective information to the learner during a training program or exercise. These included instrumented manikins, smart devices, and feedback provided by medical devices or other technologies.
- The comparator or control (C) was CPR training without corrective feedback devices, including instructor-led demonstration or feedback using static manikins or autonomous training (without instructor) using static manikins.
- The main outcomes (O) selected were CPR quality parameters, namely, the quality of chest compression–related components.
- The type of studies (S) included was randomized controlled trials (RCTs).

Studies that included participants performing CPR in real patients were excluded. Studies focused on team CPR performance were excluded. Animal studies, case reports, conference abstracts, reviews, trial protocols, and studies with incomplete or missing data were excluded. Publications prior to 2015 were excluded to minimize bias from outdated CPR guidelines and to ensure that the technology in the included studies provides corrective feedback, thus reducing heterogeneity and enhancing the reliability of the findings. No country or language restrictions were considered.

### Selection Process

The study selection process was executed using CADIMA software [35], which facilitated effective management and tracking of the selection process as well as analysis of interreviewer agreement.

Initially, a pilot analysis was conducted by 2 reviewers (AN and IJ), who independently assessed 100 abstracts. This preliminary stage aimed to evaluate the strength of agreement between reviewers and to identify and adjust any significant discrepancies in their assessment. The interrater reliability was quantified using the Cohen's κ statistic.

After refining the analysis approach, as informed by the initial pilot analysis, the reviewers transitioned to the screening phase. During this phase, titles and abstracts underwent an independent screening process conducted by the same 2 reviewers (AN and IJ). This step was crucial to ensure a thorough and unbiased selection process, and only relevant studies were considered for full-text review.

The next step was the inclusion phase, where selected papers were subjected to a comprehensive full-text analysis. This phase was expanded to include a third reviewer (CSC) to bring additional perspective and expertise, particularly in cases of initial disagreement. All 3 reviewers (AN, IJ, and CSC) independently analyzed the full texts.

In both the screening and inclusion phases, differences in opinions were resolved through in-depth discussions until a consensus was reached. During the inclusion phase, each reviewer meticulously documented the reasons for excluding papers. This step was critical for ensuring transparency and accountability in the selection process.

### Data Extraction and Outcomes

Data extraction was performed by 2 reviewers (IJ and AN) using a standardized data extraction form. CPR quality parameters were considered, namely the overall chest compression quality score. Additional parameters were extracted, including compression depth, compression frequency, chest wall recoil, and hand positioning.

Extracted data included authors, year of publication, country, study design, type of participants (laypeople or health care professionals), type of intervention, type of control, feedback device, number of participants in each group, training duration, CPR quality overall score (%), and quality of each parameter of chest compressions (%).

### Risk of Bias Assessment

The quality of the selected studies was assessed using the Cochrane risk-of-bias tool for randomized trials (RoB 2) [36] by 2 independent reviewers (AN and IJ). RoB 2 tool evaluates each study using 5 different criteria: randomization process, deviations from intended interventions, missing outcome data, measurement of the outcome, and selection of the reported result. For each item, the risk was marked as low, some concerns, and high. Uncertainties and disagreements were addressed and resolved through consensus. Studies were not blinded regarding authors, institutions, and journals. Publication bias was assessed through a funnel plot.

### Synthesis Methods

A meta-analysis and a narrative synthesis were conducted to identify associations and explore heterogeneity in the selected studies. Data were analyzed using R (version 4.3.2; R Foundation for Statistical Computing). In the meta-analysis, the mean and SD of the data were primarily used. In cases where studies presented median and IQR, these were converted into mean and SD under the assumption of normally distributed data [37]. This assumption is based on the statistical principle that, in a normally distributed sample, the mean is an approximate measure of the median, and the SD can be estimated by dividing the IQR by 1.35 [37,38]. If the mean and SD could not be estimated, those were excluded from the meta-analysis. The effect size was calculated using the mean differences with 95% CIs. The random-effects model was used because of the variation in study characteristics. Heterogeneity was studied using *I*^2^ statistic, with values below 40% representing low heterogeneity [34]. Several studies had a multiple-arm RCT design; in those cases, the relevant groups (intervention and control) were included in the pairwise comparison of intervention groups. Studies with distinct groups of adult and pediatric CPR training were included with multiple intervention groups.

Sensitivity analysis was conducted through subgroup analysis to identify the sources of heterogeneity and measure the effects within each subgroup (eg, acquisition vs retention). This analysis also determined if the performance scores varied across the subgroups. The subgroup analyses explored potential effect modifiers, including skills acquisition (assessed immediately after training), training duration (≤30 vs >30 minutes), and time elapsed after training (3 vs 9-12 months). Other subgroups were considered (eg, target audience—health care professionals vs laypeople) but were not performed due to the limited or absent number of trials available. Subgroups with enough data were used for meta-analysis.

A narrative synthesis was performed for all included studies, including those excluded from meta-analysis, following the SWiM (Synthesis Without Meta-Analysis) reporting guidelines [39]. The studies were grouped by the training-assessment interval, clearly distinguishing between skills acquisition and retention over time. When available, the main outcome selected was chest compression score. The certainty of evidence was evaluated using the GRADE (Grading of Recommendations Assessment, Development and Evaluation) approach [40].

## Results

### Search Results

**Figure 1:**
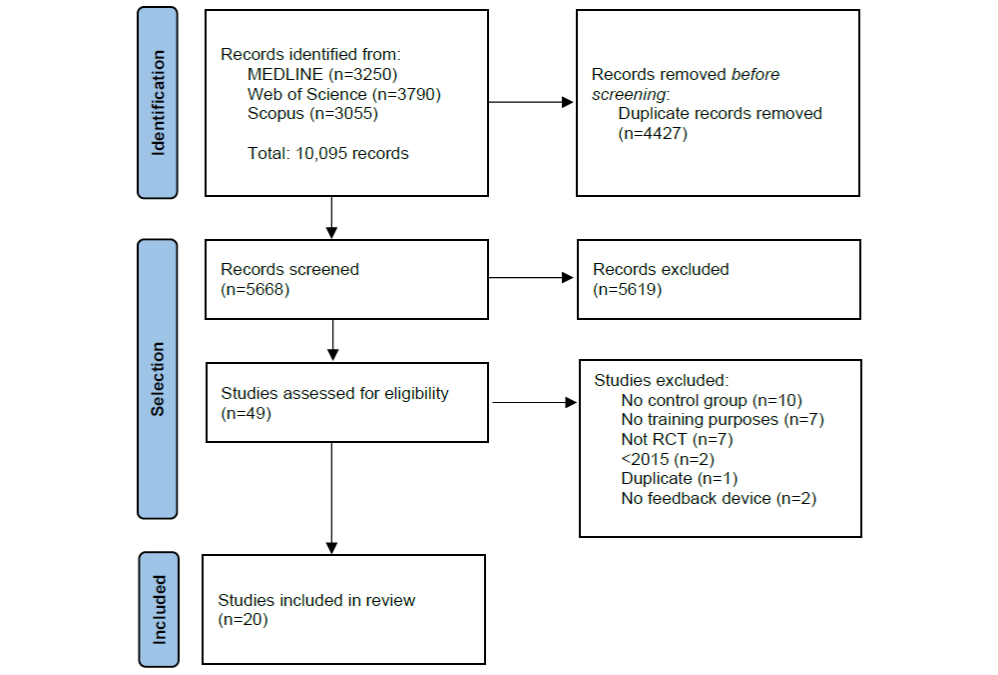
PRISMA (Preferred Reporting Items for Systematic Reviews and Meta-Analyses) flow diagram of the selection process. RCT: randomized controlled trial.

The studies’ selection process is represented in the PRISMA flow diagram [33] (Figure 1). The initial search retrieved 10,095 results, of which 4427 were duplicates. Of the remaining 5668 unique publications, 5619 were excluded after screening the title and abstract. The remaining 49 publications were screened for a full review, resulting in the 20 studies included. All included studies reported approval by an ethical committee.

Cohen's κ coefficient of agreement between the reviewers was 0.49 at the pilot screening stage, representing a fair agreement [34]. Throughout the full screening stage, the Cohen’s κ coefficient improved to 0.62, representing a good agreement. The overall percentage of agreement was 99%.

### Characteristics of Included Studies

Table 1 presents the main characteristics of the 20 included studies. Studies data were collected from 15 distinct countries. The study population is diverse, including laypeople [23,41-43], health care professionals [44-46], and lifeguards [47], among others. The sample size is heterogeneous. Although the majority of the included studies used Laerdal QCPR technology in the intervention group, the training methodologies vary substantially, with considerable differences in training duration, elapsed time to assessment, and assessment format (Table 2).

Table 2 details relevant characteristics regarding study design and outcomes. Training methodologies included short single training sessions [23,41,42,44,46,47,49,50,52,53,55,56,58,59], short recurrent practice over time [45], and long single training sessions [48,51,54,57]. The educational adjuncts in all studies were based on corrective feedback devices, although some also included video-based instructions or allied corrective feedback devices with instructor feedback [42,43,46,48,51,55,58,59]. Regarding training-assessment interval, skills acquisition assessment was not comprised in all studies, and assessment intervals for skills retention span from few weeks to several months. Skills acquisition was assessed by 14 studies [41-44,46,48-53,57-59]. In total, 6 studies presented the assessment of skills retention at 3 months [42,44,45,49,57,59], and 3 studies [45,49,59] included an assessment at 9 to 12 months. Assessment duration also varied among the studies, with most considering 2 minutes of CPR, with only 4 studies using a different assessment duration [41,47,53,55]. Assessment exercises ranged from continuous chest compression [23,41,42,45,49,56-59] to a defined number of CPR cycles [44,55]. The outcomes were mostly presented using mean and SD and median and IQR for chest compression–related parameters and overall score. Additional specifications of the included studies can be found in Multimedia Appendix 3.

**Table 1:** Characteristics of the included studies.

Study	Country	Sample	Previous training	Feedback device or data collection
Baldi et al (2017) [41]	Italy	450 Laypeople	Prior to the study: no previous training Upon entry to the study: 5-hour BLS^a^ or AED^b^ courses with 1 hour of theory and 4 hours of practice, with different training time with feedback	Training: Laerdal Resusci Anne Assessment: Laerdal Resusci Anne
Chamdawala et al (2021) [42]	United States	220 High school students	Prior to the study: regardless Upon entry to the study: 30-minute standard CPR^c^ training with an instructor	Training: Laerdal Resusci Anne Assessment: Laerdal Resusci Anne
Cortegiani et al (2017) [23]	Italy	125 High school students	Prior to the study: unknown Upon entry to the study: 30-minute standard BLS-D^d^ training with an instructor; practice until reached the minimum technical skill	Training: Laerdal Resusci Anne Assessment: Laerdal Resusci Anne
Eshel et al (2019) [48]	Israel	145 First-year medical students	Prior to the study: no previous training Upon entry to the study: 1-hour lecture and 10 academic hours of review and hands-on CPR training	Training: Laerdal Resusci Anne Assessment: Laerdal Resusci Anne
González-Santano et al (2020) [47]	Spain	30 Beach lifeguards	Prior to the study: BLS training in the previous 2 years Upon entry to the study: 12-minute session of training with at least 6 minutes of CPR	Training app: Massage cardiaque et DSA app Training device: Laerdal Resusci Anne Assessment: Laerdal Resusci Anne
Jang et al (2020) [49]	South Korea	95 University students	Prior to the study: unknown Upon entry to the study: standard BLS or AED training program	Training: BT-SEEM BT-Inc CPR training manikin Assessment: BT-CPTA BT-Inc CPR training manikin
Katipoglu et al (2021) [50]	Poland	111 First-year medical students	Prior to the study: unknown Upon entry to the study: standard BLS course	Training: Laerdal Resusci Anne Assessment: Laerdal Resusci Anne
Kim et al (2021) [51]	South Korea	64 Junior nursing students	Prior to the study: no previous training Upon entry to the study: 40-minute theoretical online lecture session and an 80-minute noncontact practice session	Training: Laerdal Resusci Anne Assessment: Laerdal Resusci Anne
Labuschagne et al (2022) [52]	South Africa	53 Final-year medical students	Prior to the study: conventional CPR training in the third year of the medical program Upon entry to the study: 1-hour CPR theoretical lesson every 2 weeks	Training: QCPR manikins Assessment: QCPR manikins
Lee et al (2023) [44]	Taiwan	90 Nurses	Prior to the study: recertification CPR program Upon entry to the study: 10-minute standardized CPR lecture and 30-minute practice	Training: Laerdal Little Anne QCPR Assessment: Laerdal Little Anne QCPR
Lin et al (2018) [45]	Canada	87 Health care providers	Prior to the study: PALS^e^ or ACLS^f^ certification within the past 2 years Upon entry to the study: standard BLS course	Training: Laerdal Resusci Anne Assessment: Laerdal Resusci Anne
Moreno et al (2021) [46]	Spain	212 Primary care staff	Prior to the study: unknown Upon entry to the study: 10-minute standardized CPR lecture and 6-minute practice	Training: Laerdal Little Anne QCPR Assessment: Laerdal Little Anne QCPR
Pavo et al (2016) [53]	Austria	326 Third-year medical students	Prior to the study: basic BLS skills Upon entry to the study: 2 hours of standard BLS (AED-BLS) training; practice until feel sufficient confidence	Training: HeartStart MRx with Q-CPR-Technology on AmbuManC manikin Assessment: AmbuManC manikins with the Ambu CPR software
Sá-Couto et al (2018) [54]	Portugal	39 Medical and engineering students	Prior to the study: no previous training Upon entry to the study: revision of CPR guidelines and algorithm	Training: CPR personal trainer on Simulaids Adult Brad Assessment: video analysis and checklist
Sarac (2017) [55]	Turkey	76 Second-year university students	Prior to the study: no previous training Upon entry to the study: 14-week first aid course with 2 weeks, 2 hours per week CPR section	Training: Laerdal Little Anne Assessment: Laerdal Resusci Anne
Smereka et al (2019) [56]	Poland	94 First-year nursing students	Prior to the study: no previous training Upon entry to the study: standard BLS course+10-minute practical training	Training: TrueCPR on an unknown training manikin Assessment: unknown training manikin
Suet et al (2020) [57]	France	61 Second-year medical students	Prior to the study: unknown Upon entry to the study: 1-day duration course with CPR training representing half of the day	Training app: iPhone app ZOLL PocketCPR on Laerdal Resusci Anne Training QCPR: Laerdal Resusci Anne Assessment: Laerdal Resusci Anne
Tanaka et al (2019) [43]	Japan	497 Laypeople	Prior to the study: regardless Upon entry to the study: instructor-led lecture followed by psychomotor practice focused on chest-compression CPR	Training: Laerdal Little Anne with QCPR Classroom Assessment: Laerdal Little Anne with QCPR Classroom
Wagner et al (2019) [58]	Austria	653 Third-year medical students	Prior to the study: unknown Upon entry to the study: revision of CPR guidelines and demonstration of the CPR algorithm by an instructor	Training: Laerdal Resusci Anne Assessment: Laerdal Resusci Anne
Zhou et al (2020) [59]	China	97 Third-year medical students	Prior to the study: regardless Upon entry to the study: 45-minute CC^g^-only CPR training program with 5 cycles of CC on manikins	Training: Laerdal Resusci Anne Assessment: Laerdal Resusci Anne

^a^BLS: basic life support.

^b^AED: automated external defibrillator.

^c^CPR: cardiopulmonary resuscitation.

^d^BLS-D: basic life support and defibrillation.

^e^PALS: pediatric advanced life support.

^f^ACLS: advanced cardiovascular life support.

^g^CC: chest compression.

**Table 2:** General design and outcomes of included studies.

Study	Intervention or control	Assessment	Training-assessment interval	Main outcomes or results
Baldi et al (2017) [41]	Control: BLS^a^ or AED^b^ course without any feedback. Intervention SF^c^: BLS or AED with a 1-minute training session with a feedback device. Intervention LF^d^: BLS or AED with a 10-minute training session with a feedback device.	1-minute chest compression session without feedback.	Postintervention	The percentage of compressions with correct depth was higher in the LF group compared to the control (75.7% vs 66.6%).
Chamdawala et al (2021) [42]	Control: practice CPR^e^ with a schoolteacher. Training duration not available. Intervention: 2-minute training session with feedback device and instructor verbal prompts if needed.	2-minute chest compression session without feedback.	Postintervention 10 weeks 28 weeks 52 weeks	The intervention group had a higher compression score (61%) than the control group (41%).
Cortegiani et al (2017) [23]	Control: 2-minute training session with an instructor. Intervention: 2-minute training session with feedback device.	2-minute chest compression session without feedback.	7 days	The intervention group had a higher compression score (90%) than the control group (67%).
Eshel et al (2019) [48]	Control: academic course with 10 hours of CPR training with an instructor. Intervention: academic course with 10 hours of CPR training with instructor and feedback device.	CPR OSCE^f^ evaluation at the end of the course. Assessment duration not available.	Postintervention	The intervention group had a higher chest compression fraction (57%) than the control group (49%).
González-Santano et al (2020) [47]	Control: at least, 6-minute training session with instructor. Intervention—app: at least, a 6-minute training session with the app. Intervention—feedback device: at least, a 6-minute training session with a feedback device.	3-minute CPR session without feedback.	Between 7 and 15 days	The percentage of compressions with correct depth was higher in the intervention group (68.2% vs 30.8%).
Jang et al (2020) [49]	Control: four 2-minute sessions of CPR without feedback. Intervention: four 2-minute sessions of CPR with a feedback device.	2-minute chest compression session without feedback.	Postintervention 3 months 6 months 9 months	The intervention group presented higher percentage of adequate chest compression depth than the control group (51% vs 26.9%).
Katipoglu et al (2021) [50]	Control: 15-minute CPR training session without feedback. Intervention: 15 minutes of CPR training with a feedback device.	2-minute CPR session with feedback for the intervention group on postintervention assessment and without feedback on 1-month follow-up.	Postintervention 1 month	The intervention group presented better depth on chest compressions than the control group (50 vs 39 mm).
Kim et al (2021) [51]	Control: 80-minute practice session with nurse. Intervention: 80-minute practice session with feedback device and feedback from nurse.	Not available.	Postintervention 4 weeks	The intervention group had a slightly higher score in CPR performance (37.23) than the control group (33.06).
Labuschagne et al (2022) [52]	Control: 10-minute standard CPR training without an instructor. Intervention: 10-minute CPR training with a feedback device.	2-minute CPR session without feedback	Postintervention	Similar results in both groups.
Lee et al (2023) [44]	Control: 30-minute standard CPR training with an instructor. Intervention: 30-minute CPR training with a feedback device.	5 cycles of CPR	Postintervention 12 weeks	The intervention group had significantly higher chest compression scores when compared to the control group.
Lin et al (2018) [45]	Control: standardized AH^g^ BLS course with an instructor. Training duration not available. Intervention: 2-minute training session with a feedback device, at least once a month.	2-minute chest compression session without feedback.	3 months 6 months 9 months 12 months	The intervention group had a greater proportion of participants with excellent CPR (54.3%) than the control group (14.6%).
Moreno et al (2021) [46]	Control: CPR training with an instructor. Intervention: CPR training with feedback device and instructor.	2-minute CPR session without feedback.	Postintervention 6 months	The main outcome improved with statistical significance in the intervention group with respect to control.
Pavo et al (2016) [53]	Control: CPR training session with feedback provided by a team member. Training duration not available. Intervention: CPR training session with feedback provided by feedback device. Training duration not available.	8-minute, 2-rescuer CPR session without feedback.	Postintervention	The quality of CPR in control and intervention groups was similar.
Sá-Couto et al (2018) [54]	Control: 1-hour training session with an instructor. Intervention: 1-hour training session with a feedback device.	2-minute BLS algorithm session without feedback, rated with a checklist.	1 week	Both groups presented identical mean differences for the total score, with no statistical difference.
Sarac (2017) [55]	Control: first aid course with instructor. Intervention—real time: 10 CPR sets with real-time feedback provided by feedback device and instructor when necessary. Intervention—report: 10 CPR sets with instructor feedback and printed report of CPR skills after training.	10 sets of CPR without feedback.	4 weeks	Both interventions performed better in some compression skills than the control group.
Smereka et al (2019) [56]	Control: 30-minute autonomous training session without a feedback device. Intervention: 30-minute training session with a feedback device.	2-minute chest compression session without feedback.	1 month	The intervention group is associated with better CPR skills 1 month after the training period.
Suet et al (2020) [57]	Control: half-day CPR training session with an instructor. Intervention—app: half-day CPR training session with an instructor and guided iPhone app PocketCPR. Intervention—QCPR: half-day CPR training session with a feedback device.	2-minute chest compression session without feedback.	Postintervention 3 months	QCPR group had a higher median compression score (72%) than the control group (22.5%).
Tanaka et al (2019) [43]	Control: standard CPR training with an instructor. Training duration not available. Intervention: CPR training with instructor and feedback device. Training duration not available.	2-minute CPR session without feedback.	Postintervention	The intervention group improved at 39%, while the control group improved at 20%.
Wagner et al (2019) [58]	Control: 2-minute training session with an instructor. Intervention—only feedback device: 2-minute training session with a feedback device. Intervention—feedback device and instructor: 2-minute training session with feedback from instructor seen on feedback device.	2-minute chest compression session without feedback.	Postintervention	Both interventions were similar for total compression score (DF^h^: 87.2%; IDF^i^: 93.2%) but significantly better than the control group (77.3%).
Zhou et al (2020) [59]	Control: 30-minute training session with an instructor. Intervention: 30-minute training session with feedback device and instructor.	2-minute chest compression session without feedback.	Day 1 (postintervention) Day 3 (postintervention 2) Day 7 (postintervention 3) 3 months 12 months	Similar results in both groups.

^a^BLS: basic life support.

^b^AED: automated external defibrillator.

^c^SF: short feedback.

^d^LF: long feedback.

^e^CPR: cardiopulmonary resuscitation.

^f^OSCE: Objective Structured Clinical Examination.

^g^AHA: American Heart Association.

^h^DF: device feedback.

^i^IDF: instructor and device feedback.

### Risk of Bias in Studies

The risk of bias was assessed in all 20 RCTs. Among the studies included, 40% (n=8) presented a high risk of bias [42,43,46,48,49,52,55,57], and 50% (n=10) presented a low risk of bias [23,41,44,45,50,51,53,56,58,59]. Table 3 illustrates the risk of bias in each domain (D1-D5), based on RoB 2 [36], along with the overall risk of bias.

Across all domains, the randomization process was the primary reason for the risk of bias due to no information or absence of random allocation sequence or no information or absence of allocation sequence concealment. Studies were assessed to have an overall “high risk” of bias if there were at least 1 “high risk” in any of the criteria (D1-D5).

**Table 3:** Risk of bias—randomization process (D1), deviations from intended interventions (D2), missing outcome data (D3), measurement of the outcome (D4), and selection of the reported result (D5).

Study ID	D1	D2	D3	D4	D5	Overall
Baldi et al (2017) [41]	+^a^	+	+	+	+	+
Chamdawala et al (2021) [42]	+	±^b^	–^c^	+	+	–
Cortegiani et al (2017) [23]	+	+	+	+	+	+
Eshel et al (2019) [48]	–	+	+	+	+	–
González-Santano et al (2020) [47]	+	+	±	+	+	±
Jang et al (2020) [49]	±	+	–	+	+	–
Katipoglu et al (2021) [50]	+	+	+	+	+	+
Kim et al (2021) [51]	+	+	+	+	+	+
Labuschagne et al (2022) [52]	–	+	+	+	–	–
Lee et al (2023) [44]	+	+	+	+	+	+
Lin et al (2018) [45]	+	+	+	+	+	+
Moreno et al (2021) [46]	±	+	–	+	+	–
Pavo et al (2016) [53]	+	+	+	+	+	+
Sá-Couto et al (2018) [54]	±	+	+	+	+	±
Sarac (2017) [55]	–	+	+	+	+	–
Smereka et al (2019) [56]	+	+	+	+	+	+
Suet et al (2020) [57]	+	+	+	–	+	–
Tanaka et al (2019) [43]	±	+	–	+	+	–
Wagner et al (2019) [58]	+	+	+	+	+	+
Zhou et al (2020) [59]	+	+	+	+	+	+

^a^**+**: low risk.

^b^**±**: some concerns.

^c^**–**: high risk.

### Synthesis of CPR Quality

**Figure 2:**
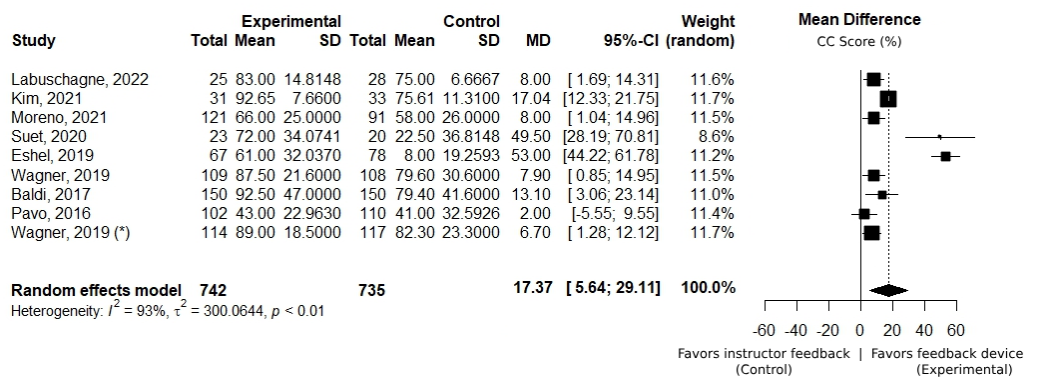
Forest plot presenting a mean difference of overall chest compression (CC) score (%) between the intervention (with feedback device) and control groups (instructor feedback). Positive values favor the use of feedback devices. Data collected immediately after training (skills acquisition assessment) [41,46,48,51-53,57,58]. * represents pediatric cardiopulmonary resuscitation training.

The skills acquisition subgroup included 8 studies [41,46,48,51-53,57,58] involving 1477 participants. Meta-analysis results revealed high heterogeneity (*I*^2^>75%, as illustrated in Figure 2). All studies presented a positive mean difference for chest compression score, with an average of 17.37%, favoring the use of corrective feedback devices.

**Figure 3:**
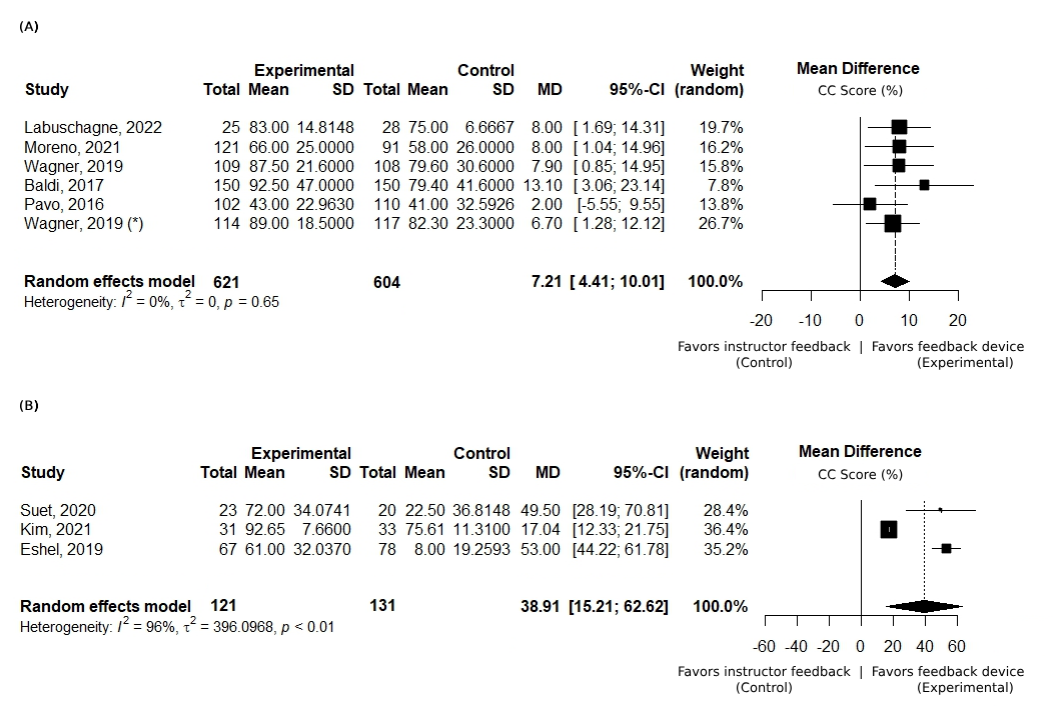
Overall chest compression (CC) score: subgroup analysis based on the duration of training. Set of forest plots presenting a mean difference of overall CC score (%) between the intervention (with feedback device) and control groups (instructor feedback). Positive values favor the use of feedback devices. Data collected immediately after training (skills acquisition assessment). Subgroup analysis: (A) studies with less than 30 minutes of training time [41,46,52,53,58] and (B) studies with more than 30 minutes of training time [48,51,57]. * represents pediatric cardiopulmonary resuscitation training.

Within the skills acquisition subgroup, further analysis was conducted to examine the duration of training. Five studies, involving a total of 1225 participants, focused on training sessions shorter than 30 minutes [41,46,52,53,58]. These studies uniformly reported a favorable mean difference in chest compression score, with an average improvement of 7.21%, attributed to the use of corrective feedback devices (as shown in Figure 3A). These studies exhibited low heterogeneity (*I*^2^=0%), underscoring the consistency of the positive effects observed. Three studies, involving 252 participants, reported on training sessions with more than 30 minutes [48,51,57] with high heterogeneity (*I*^2^>75%). Despite the observed variability, all studies consistently presented a substantial mean improvement in chest compression score, averaging 38.91% (as shown in Figure 3B), underscoring the impact of using corrective feedback devices in longer training sessions.

**Figure 4:**
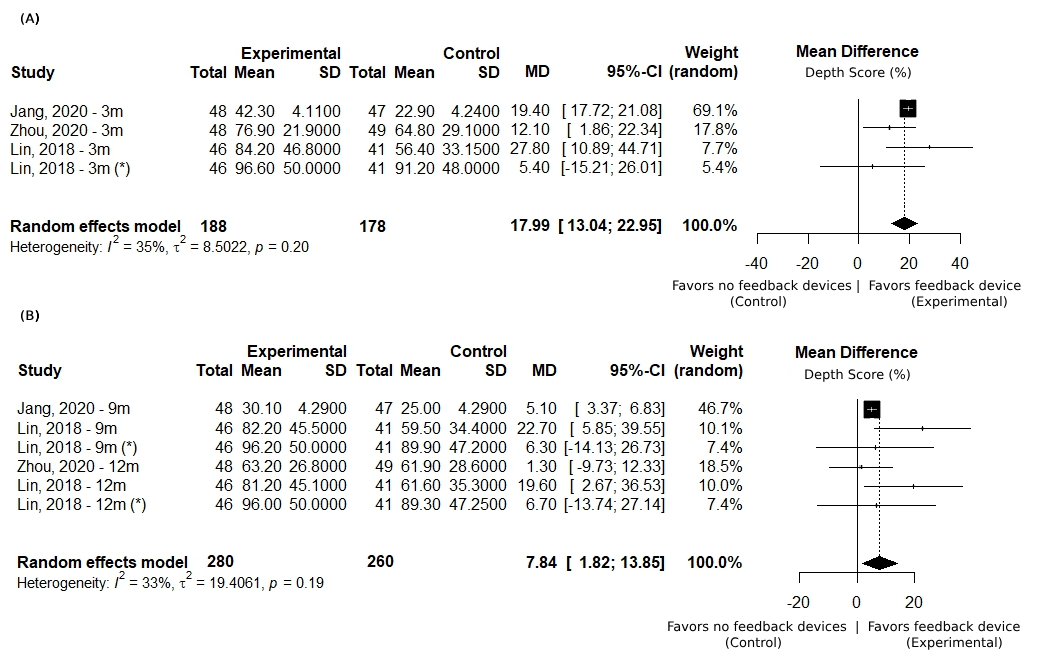
Chest compression depth: subgroup analysis based on elapsed time after training. Set of forest plots presenting a mean difference of depth score (%) between the intervention (with feedback device) and control groups (instructor feedback). Positive values favor the use of feedback devices. Subgroup analysis for different periods of retention: (A) 3 months and (B) 9 to 12 months [45,49,59]. * represents pediatric cardiopulmonary resuscitation training.

Skills retention at 3 months was examined in 3 studies [45,49,59], involving 366 participants, with low heterogeneity (*I*^2^=35%). These studies collectively showed a positive mean difference in depth scores, averaging 17.99%, favoring the use of corrective feedback devices (as illustrated in Figure 4A). For skills retention between 9 and 12 months, 3 studies [45,49,59] were analyzed, revealing low heterogeneity (*I*^2^=33%). All studies reported a positive mean difference in depth score, with an average improvement of 7.84%, favoring the use of corrective feedback devices (as shown in Figure 4B).

Other meta-analyses were conducted to investigate potential sources of heterogeneity, including a subgroup analysis based on risk of bias and study population; however, no significant results were observed. This meta-analysis did not assess the quality of chest recoil, hand positioning, and compression frequency due to insufficient data or challenges in extracting mean and SD values. Certainty of evidence was assessed using the Grading of Recommendations Assessment, Development and Evaluation approach, revealing a very low to low certainty (as shown in Table 4). Publication bias was not detected through the analysis of funnel plots (Multimedia Appendix 4).

**Table 4:** Certainty of evidence (GRADE approach).

	Studies, n	Study design	Risk of bias	Inconsistency	Indirectness	Imprecision	Certainty
CPR^a^ skills acquisition: chest compressions score (%)	8	RCT^b^	Serious	Serious^c^	Not serious^d^	Serious^e^	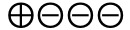 Very low
CPR skills retention: compressions depth score (%)	3	RCT	Serious	Not serious	Not serious	Serious^e^	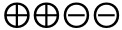 Low

^a^CPR: cardiopulmonary resuscitation.

^b^RCT: randomized controlled trial.

^c^High heterogeneity was identified between studies leading to inconsistency.

^d^Outcome measures were directly associated with the research question. The majority of the corrective feedback devices provided the required outcomes.

^e^High SDs in most studies.

## Discussion

### Principal Findings

This paper critically evaluates the effectiveness of corrective feedback devices in CPR training for both laypeople and health care professionals. While several reviews support the use of feedback devices for CPR training [29-32], some studies report inaccuracies related to the use of these devices, namely an overestimation of chest compression depth [60,61]. To the best of our knowledge, there is no recent and thorough systematic review and meta-analysis specifically evaluating the effects of CPR training with corrective feedback devices on the skills acquisition and retention among both laypeople and health care professionals. Existing reviews often rely on outdated guidelines [29,30], or encompassed a broader scope, such as the use of feedback devices in real clinical settings [29-31].

Notably, reviews prior to 2015 often did not differentiate between devices providing guidance and those offering corrective feedback, a distinction introduced in 2015 CPR guidelines [28]. One of the earliest systematic reviews showed evidence favoring the use of CPR feedback devices [29]; however, it included studies based on outdated guidelines and “prompt” devices.

A more recent review also reported that feedback devices promote better chest compressions [30]. However, this review included nonrandomized trials, studies based on outdated guidelines, and studies performed in real settings (human studies), increasing the heterogeneity and potentially reducing the quality of the review. Subsequent reviews [31,32] recommended the use of feedback devices but did not include a meta-analysis [31] and focused on specific groups—health care professionals [31] and laypeople [32]. Additionally, these reviews included studies using devices that lacked corrective feedback.

This systematic review and meta-analysis aim to bridge the identified gaps by examining the impact of corrective feedback devices on the quality of chest compressions during CPR training across diverse groups. This review exclusively focuses on studies using corrective feedback devices for skills acquisition and retention, within simulated or training environments, targeting both laypeople and health care professionals. The rigorous search strategy and selection process ensured a comprehensive and inclusive examination, capturing all pertinent studies within the scope of this review.

The risk of bias assessment revealed a general trend of overestimated effects due to methodological flaws, with few studies adhering to CONSORT (Consolidated Standards of Reporting Trials) reporting guidelines [62]. Poorly designed studies may yield larger effect estimates when compared to those with rigorous methodologies, potentially compromising the robustness and reliability of the findings. Several studies included in the meta-analysis were noted to have some concerns or high risk of bias, which may influence the overall findings. These were not excluded to maintain an adequate number of studies under analysis, considering that all of those are RCT, which, per se, represent a superior level of evidence [63].

The majority of studies included in this review featured intervention groups that conducted training autonomously or through self-training without direct instructor involvement. However, some studies did incorporate a degree of instructor mediation within the intervention groups. In these cases, the corrective feedback device remained the central component of the intervention, with the instructors’ roles primarily confined to presenting the metrics generated by these devices.

The presence or absence of an instructor in the intervention groups was not considered a criterion for inclusion in this review, which may have introduced a degree of heterogeneity. The varied outcomes and measurement approaches revealed a notable absence of standardized research protocols in this field. Specifically, for identical chest compression parameters, multiple approaches for measuring outcomes were identified. Adoption of standardized protocols and assessments in future research could greatly contribute to accurately extract intervention effects, allowing a more reliable and precise comparison of results.

The conducted meta-analysis revealed significant heterogeneity. Several factors may have contributed to this variability: the quality of the studies included; the variable protocol designs and methodologies used across these studies, including variations in training methods and assessment procedures; the diversity of the outcomes measured; the presence of nonreporting bias, where studies showing nonsignificant or minimal effect sizes might be underreported; and intrinsic heterogeneity due to a wide range of variables identified as relevant to the analysis.

Despite significant heterogeneity among some study results, this meta-analysis provides insights into the positive impact of corrective feedback devices on CPR training. As illustrated in Figure 2, there is an overall positive mean difference between the control and intervention groups, suggesting a positive impact of corrective feedback devices in enhancing skills acquisition and improving the quality of chest compressions. Notably, the duration of training plays a critical role in this improvement. Shorter training sessions show a reduced mean difference in chest compression scores between groups, indicating that extended exposure to corrective feedback devices yields better training outcomes. This observation is further supported by the consistency of results across studies with training durations of less than 30 minutes, which exhibit low heterogeneity.

In the analysis of skills retention over time, the depth of chest compressions emerged as the only parameter suitable for inclusion in the meta-analysis. Examining this specific parameter revels that the difference between the control group and the intervention group considerably decreases, when comparing data from the 3-month and 9- to 12-month subgroups, accompanied by an increase in heterogeneity (as illustrated in Figure 4). This trend suggests that, while the immediate benefits of using corrective feedback devices to enhance the quality of chest compression depth are clear, these benefits tend to converge over time, leading to less pronounced differences between groups and greater variability in study findings.

The maximum follow-up time analyzed in this review was 12 months due to the lack of reliable studies with longer follow-up periods. More robust studies with extended follow-up times are needed, especially considering that the recommended interval for recertification is 24 months. The studies with health care professionals [44-46] were too few to allow a comparison with studies focusing on laypeople; however, the results suggest a similar impact of corrective feedback devices in both groups. Future work could focus on exploring the differences in CPR skills acquisition and retention between these groups.

Regarding the corrective feedback devices used, the majority were Laerdal products, including full-body simulators or torsos. It was unclear whether these devices provided real-time feedback or presented a summary of the CPR quality at the end of each training session. While real-time feedback could enable immediate correction of maneuvers, it might also serve as a distraction during training sessions. This analysis, to our knowledge, has not been studied and could be an important topic for future research.

The majority of the studies included in this review revealed that the use of corrective feedback devices during CPR training led to significant enhancements in 1 or more key chest compression parameters. Although the improvements across all parameters were not uniform, the evidence strongly supports that corrective feedback devices contribute to an overall improvement in CPR skills acquisition among both laypeople and health care professionals, especially when exposed to longer training sessions. Longitudinal analysis of skills retention indicates that the long-term benefits become less distinct, especially after 3 months. These results are consistent with the findings from a previous systematic review [32].

The contribution of corrective feedback devices is significant not only for more objective and standardized training but also for enabling more frequent practice, without being dependent on the presence of an instructor. This facilitates continuous improvement and broader preparation for performing CPR.

### Limitations

This review is not without limitations. The high heterogeneity, variations in the study designs, and generally low quality of the included studies challenge the validity and generalization of conclusions. Additionally, this review only included RCTs, which may hinder relevant conclusions resulting from other designs. The exclusion of studies published before 2015 may have also omitted relevant data. Moreover, the use of different tools across studies to evaluate training outcomes introduces further variability, potentially affecting the judgment of findings.

### Conclusions

This review supports the use of corrective feedback devices in CPR training as a beneficial complement or surrogate to traditional training methods (instructor-based feedback). Corrective feedback devices offer objective insights that can enhance immediate skills acquisition and may contribute to long-term retention. However, these findings must be interpreted with caution due to the significant variation in study designs and outcomes, along with the overall low quality of existent studies, highlighting the urgent need for well-designed, high-quality studies with standardized protocols.

## Appendix

Multimedia Appendix 1 PRISMA (Preferred Reporting Items for Systematic Reviews and Meta-Analyses) 2020 checklist.

Multimedia Appendix 2 Search strategy.

Multimedia Appendix 3 Study characteristics.

Multimedia Appendix 4 Publication bias—funnel plot.

## Abbreviations

BLS: basic life support
CONSORT: Consolidated Standards of Reporting Trials
CPR: cardiopulmonary resuscitation
OHCA: out-of-hospital cardiac arrest
PICOS: participants, intervention, control, outcomes, and studies
PRISMA: Preferred Reporting Items for Systematic Reviews and Meta-Analyses
RCT: randomized controlled trial
SCA: sudden cardiac arrest
SWiM: Synthesis Without Meta-Analysis

## Data Availability

The search strategy is provided in Multimedia Appendix 2. The datasets generated and analyzed during this study are available from the corresponding author on reasonable request.
