# Supplementary material for: Influence of Training With Corrective Feedback Devices on Cardiopulmonary Resuscitation Skills Acquisition and Retention: Systematic Review and Meta-Analysis
Source: JMIR Med Educ. 2024 Dec 19;10:e59720. doi: 10.2196/59720 (PMC11695954; doi:10.2196/59720)
Supplement: Multimedia Appendix 2 [file mededu_v10i1e59720_app2.pdf]

Multimedia Appendix 2 – Search Strategy

General Strategy

Publications from January 2015 to December 2023. MEDLINE, Web of Science and Scopus.
1. Cardiopulmonary resuscitation [tab]<br>2. CPR [tab]<br>3. Basic life support [tab]<br>4. BLS [tab]<br>5. Resuscitation [tab]<br>**6. 1 OR 2 OR 3 OR 4 OR 5**<br><br>7. Training [tab]<br>8. Feedback [tab]<br>9. Manikin* [tab]<br>10. Device* [tab]<br>11. Prompt* [tab]<br>12. Audiovisual [tab]<br>13. Technology [tab]<br>14. Simulat* [tab]<br>**15. 7 OR 8 OR 9 OR 10 OR 11 OR 12 OR 13 OR 14**<br><br>16. Quality [tab]<br>17. Performance [tab]<br>18. Compression* [tab]<br>19. Acquisition [tab]<br>20. Retention [tab]<br>**21. 16 OR 17 OR 18 OR 19 OR 20**<br><br>**22. 6 AND 15 AND 21**

MEDLINE – PubMed

Search	Query
#23	Search: #22 AND 2015:2023[dp]
#22	Search: #6 AND #15 AND #21
#21	Search: #16 OR #17 OR #18 OR #19 OR #20
#20	Search: Retention[Title/Abstract]
#19	Search: Acquisition[Title/Abstract]
#18	Search: Compression*[Title/Abstract]
#17	Search: Performance[Title/Abstract]
#16	Search: Quality[Title/Abstract]
#15	Search: #7 OR #8 OR #9 OR #10 OR #11 OR #12 OR #13 OR #14
#14	Search: Simulat*[Title/Abstract]
#13	Search: Technology[Title/Abstract]
#12	Search: Audiovisual[Title/Abstract]
#11	Search: Prompt*[Title/Abstract]
#10	Search: Device*[Title/Abstract]
#9	Search: Manikin*[Title/Abstract]
#8	Search: Feedback[Title/Abstract]
#7	Search: Training[Title/Abstract]
#6	Search: #1 OR #2 OR #3 OR #4 OR #5
#5	Search: Resuscitation[Title/Abstract]
#4	Search: BLS[Title/Abstract]
#3	Search: Basic life support[Title/Abstract]
#2	Search: CPR[Title/Abstract]
#1	Search: Cardiopulmonary resuscitation[Title/Abstract]

Web of Science

Query: ((TS=(Cardiopulmonary resuscitation) OR TS=(CPR) OR TS=(Basic life support) OR TS=(BLS) OR TS=(Resuscitation)) AND (TS=(Training) OR TS=(Feedback) OR TS=(Manikin*) OR TS=(Device*) OR TS=(Prompt*) OR TS=(Audiovisual) OR TS=(Technology) OR TS=(Simulat*)) AND (TS=(Quality) OR TS=(Performance) OR TS=(Compression*) OR TS=(Acquisition) OR TS=(Retention))) AND DOP=(2015/2023)

Scopus

Query: ((TITLE-ABS(Cardiopulmonary resuscitation) OR TITLE-ABS(CPR) OR TITLE-ABS(Basic life support) OR TITLE-ABS(BLS) OR TITLE-ABS(Resuscitation)) AND (TITLE-ABS(Training) OR TITLE-ABS(Feedback) OR TITLE-ABS(Manikin*) OR TITLE-ABS(Device*) OR TITLE-ABS(Prompt*) OR TITLE-ABS(Audiovisual) OR TITLE-ABS(Technology) OR TITLE-ABS(Simulat*)) AND (TITLE-ABS(Quality) OR TITLE-ABS(Performance) OR TITLE-ABS(Compression*) OR TITLE-ABS(Acquisition) OR TITLE-ABS(Retention))) AND PUBYEAR > 2014
