# Supplementary material for: Influence of Training With Corrective Feedback Devices on Cardiopulmonary Resuscitation Skills Acquisition and Retention: Systematic Review and Meta-Analysis
Source: JMIR Med Educ. 2024 Dec 19;10:e59720. doi: 10.2196/59720 (PMC11695954; doi:10.2196/59720)
Supplement: Multimedia Appendix 3 [file mededu_v10i1e59720_app3.pdf]

Multimedia Appendix 3. Study characteristics.

Author	Year Published	Country	Study Design	Population		Previous Training	Intervention Description	Feedback Device		Outcomes	
				N	Participants			Training	Assessment	Assessment-Training Interval	Data
Cortegiani et al [1]	2017	Italy	Randomized Trial	125	Secondary School Students	**Previous:** unknown<br><br>**Upon study**: a trained instructor performed a 30-minute interactive frontal lesson about cardiac arrest and BLS-D. students of both groups started to practice until they reached a minimum sufficient technical skill to perform a 2-minute training session according to the instructor in charge.	**Standard Feedback from instructor group**: received feedback on their practice during the 2-minute training session according to instructor's opinion. At the end of the session, all participants received a feedback on their performance based on the instructor's judgment.<br><br>**QCPR group**: training by the instructor on chest compressions using a mannequin connected to a personal computer running specific software proving real-time electronic feedback about CPR quality. Practice until they reached a minimum sufficient technical skill to perform a 2-minute training session according to the instructor in charge. Performed a 2-minute training session taking into account the real-time data of the software trying to correct themselves to reach the high-quality targets. At the end of the session, all participants received a feedback on their performance: in the QCPR group the feedback was based on results from the software.<br><br>**Assessment:** 2-minute chest compression session without feedback neither from the software nor from the instructor.	**Standard Feedback from instructor group:** Laerdal Resusci Anne<br><br>**QCOR Group:** Laerdal Resuscie Anne QCPR with Wireless SkillReporter	Laerdal Resuscie Anne QCPR with Wireless SkillReporter for data collection	7 days	**Primary:** compression score calculated by Laerdal QCPR<br><br>**Secondary:** 1) compression rate 2) compression depth 3) percentage of compressions with complete chest release 4) percentage of compressions with correct hand position.<br><br>**Other:** 5) overall dichotomous judgment from the instructor who judged students' performance (adequate/not adequate); 6) proportion of students reaching a compression score of 75% (minimum score for an "Advanced CPR Performer" according to manufacturer's indications and confirmed by consensus among authors) and 90%; 7) proportion of students reaching a compression score of 90%; 8) 1–5 Likert-scale score from participants investigating the degree of their comfort and how they enjoyed the overall experience
Lin et al [2]	2018	Canada	Randomized Controlled Trial	87	Healthcare Providers working in the Emergency Department	**Previous**: Pediatric healthcare providers (nurses, residents, respiratory therapists) and Pediatric Advanced Life Support (PALS), or Advanced Cardiac Life Support (ACLS) certification within the past 2 years.<br><br>**Upon study**: each participant had taken an initial BLS course to ensure equal opportunity for CPR skill acquisition upon entry into the study.	**Control Group:** Standardized AHA BLS course only. Participants in the control group received the standardized AHA BLS course only, led by certified BLS instructors 12 months after taking the initial BLS course. During the course, participants practiced without real-time feedback and the course instructor evaluated the CPR quality with visual assessment for chest compression (CC) depth and recoil and a stop-watch for CC rate.<br><br>**Intervention Group:** access to a CPR training unit and received just-in-time CPR training in a longitudinal fashion in the ED. The training unit also provided feedback on all CPR metrics during compressions. At the end of each CPR practice event, the feedback device provided terminal feedback for the learner on the quality of CPR. Healthcare providers in the intervention group practiced continuous CCs for 2 min on both adult and infant-sized mannequins with real-time feedback. All participants in the intervention group were required to practice at least once a month with no maximum for the number of times they were permitted to practice.<br><br>**Assessment:** Assessment sessions were run in the same setting as the practice sessions. All participants were assessed at baseline and every 3 months during the study.	**Control Group**: Laerdal Resusci Anne, Laerdal Resusci Baby<br><br>**Intervention group:** Laerdal Resusci Anne with SimPad SkillReporter, Laerdal Resusci Baby with SimPad SkillReporter	Laerdal Resusci Anne with SimPad SkillReporter for data collection<br><br>Laerdal Resusci Baby with SimPad SkillReporter for data collection	Baseline<br>3 months<br>6 months<br>9 months<br>12 months	**Primary:** "excellent CPR", defined as achieving at least 90% of all AHA standards for CC depth, rate and recoil for each individual criterion.<br><br>**Secondary:** (i) Percentage of compression depth > 50 mm for adult/child and compression depth > 40 mm for infant, (ii) Percentage of CC with rate of 100–120/min, and (iii) Percentage of CC with complete recoil.
Chamdawala et al [3]	2021	USA	Randomized Controlled Trial	**220**	High school Students	**Previous**: All students were eligible to participate in our study, regardless of their prior experience ortraining in CPR.<br><br>**Upon study**: The next day, all study participants received standard CPR training, which consisted of a school teacher-facilitated 30-minute theoretical and practical group training session, using 'The CPR in Schools Training Kit' endorsed by the AHA.	**Standard Group:** practice CPR on the inflatable manikin under the supervision of the school teacher.<br><br>**Feedback Group**: FG performed two minutes of compression-only CPR training on the Resusci Anne QCPR manikin. The research assistant (RA)<br>provided verbal prompts if the student had difficulty interpreting the<br>feedback device.<br><br>**Assessment:** Within the two to three days following the intervention, we tested students from both groups by having them complete the same questionnaire and perform two minutes of compression-only CPR on the feedback manikin;	**Standard Group:** none. Inflatable manikin.<br><br>**Feedback Group**: Resusci Anne QCPR (Laerdal Medical Corporation, USA) with SimPad with SkillReporter	Resusci Anne QCPR (Laerdal Medical Corporation, USA) with SimPad with SkillReporter	0 weeks<br>10 weeks<br>28 weeks<br>52 weeks	**Primary:** compression score<br><br>**Secondary:** (1) participants achieving "Advanced CPR Performer" status, defined as an overall CS of 75% according to the manufacturer's guidelines; (2) individual component CPR metrics such as mean compression depth and rate, and percentage of the time the student used correct hand position and allowed for full chest recoil; (3) theoretical knowledge and self-perceived comfort in performing CPR in real-life, collected via the questionnaire during each follow-up testing session.
Moreno et al [4]	2021	Spain	Randomized Controlled Trial	**212**	Primary care staff	**Previous**: unknown<br><br>**Upon study**: a session was held joint 10-minute theory on quality CPR, by an accredited life support instructor basic (SVB), to all participants. Subsequently, All of them followed the 6-minute individualized and isolated practical training with an instructor and an Annie QCPR® manikin (Laerdal).	**Group 1**: the participant received corrections from the instructor while both the instructor and the participant visualized in the laptop monitor the quality of the compressions thoracic and ventilation;<br><br>**Group 2**: the participant received corrections from the instructor while the instructor viewed the quality of chest compressions and ventilations on the laptop monitor, but not the participant;<br><br>**Control Group**: the participant received corrections from the instructor, but neither the instructor nor the participant saw the quality of chest compressions and ventilations on the laptop monitor.<br><br>**Training**: a session was held joint 10-minute theory on quality CPR, by an accredited life support instructor basic (SVB), to all participants. Subsequently, All of them followed the 6-minute individualized and isolated practical training with an instructor and an Annie QCPR® manikin (Laerdal). 1) participants initially performed 2 minutes of CPR during which the quality of the maneuvers was recorded; 2) 2 minutes of CPR were subsequently performed with a training intervention by the instructor; and 3) finally the participants were able to perform another 2 minutes of CPR recorded by the program, without the intervention of the instructor<br><br>**Assessment**: 2 minutes of CPR recorded by the program, individually and isolated, without receiving corrections from the instructor or viewing the feedback.	**Group 1**: Little Anne QCPR system equipped with Skill Reporter wireless software (Laerdal)<br><br>**Group 2:** Little Anne QCPR system equipped with Skill Reporter wireless software (Laerdal)<br><br>**Control Group:** Little Anne QCPR system	Little Anne QCPR system equipped with Skill Reporter wireless software (Laerdal)	Post Intervention<br>6 months	Primary: compression score<br><br>Secondary: all measurements of compression quality, ventilation and chest expansion fraction, measured directly by the Skillreporter® program (Laerdal) - Compression score, Ventilation score, Compression fraction score, Number of compressions with correct decompression, Average compression depth, Number of compressions with adequate depth, Compression number with appropriate rhythm, Medium compression rhythm<br>Ventilation score, Number of ventilations with adequate volume, Average ventilation volume, Medium ventilation rate
Suet et al [5]	2020	France	Randomized Trial	**61/55**	Second-year medical students on basic emergency procedures	**Previous**: unknown<br><br>**Upon study:** For each student involved, the training session had a 1-day duration and CPR training represented half of the day.	**Control group:** traditional ECM training method. The trainer interacted with students by performing initially the task then providing continuous feedback and immediately correcting errors made by students performing the tasks themselves.<br><br>**Group 2**: training was done in the presence of the trainer but was guided by a feedback method, namely the iPhone App PocketCPR (ZOLL Medical, Chelmsford, MA).<br><br>**Group 3:** used the Resusci Anne QCPR system equipped with Skill Reporter wireless software (Laerdal).<br><br>**Training:** For each student involved, the training session had a 1-day duration and CPR training represented half of the day. The ECM part of the training session was nearly 90 min, and each student performed the procedural task several times for a total of 5–8 min. The manikin used was a Resusci Anne.<br><br>**Assessment:** perform a 2-min ECM without interruption.	**Control Group:** none. Resusci Anne manikin<br><br>**Group 2:** iPhone App PocketCPR (ZOLL Medical, Chelmsford, MA) on Resusci Anne manikin<br><br>**Group 3**: Resusci Anne QCPR system equipped with Skill Reporter wireless software (Laerdal)	Resusci Anne QCPR system equipped with Skill Reporter wireless software (Laerdal) for data collection	0 days (post-intervention)<br>3 months	**Primary:** overall quality of ECM (compression score) at 3 months after initial training.<br><br>**Secondary:** 5 different components of compression score analyzed separately, evaluation of satisfaction by a Kirkpa-trick level 1 questionnaire distributed at the end of the initial session.

Kim et al [6]	2021	South Korea	Randomized Controlled Trial	64	Junior nursing students	**Previous**: Exclusion criteria were (i) students with basic life support (BLS) certifications and (ii) students completed or taking any BLS trainings using any real-time feedback devices.<br><br>**Upon study**: The training consisted of a 40-min theoretical online lecture session and an 80-min non-contact practice session.	**Control group:** 40-min theoretical online lecture session and the 80-min face-to-face practice session with a training nurse but without real-time feedback from the smart devices. A training nurse observed and guided students' performance and provided feedback through face-to-face education. After completing each CPR performance, the students reviewed the details of their overall performance from the records of cameras in the debriefing session.<br><br>**Experimental group:** 40-min theoretical online lecture session and an 80-min non-contact practice session which used the real-time on-screen feedback devices (SkillGuide from Laerdal Medical) displaying the detailed degrees of compression depth, complete or incomplete release, compression rate, time, and hand placement continuously during the CPR performance. A training nurse can observe the student directly through a glass window, grasp facial expressions and detailed movements through two cameras, identify the accuracy of a student's CPR practice in real time through a monitor linked to a smart device, and educate the student on the CPR process and give feedback through a microphone. After completing each CPR performance, the debriefing session allowed students to recognize their overall performance and the degrees of performance accuracy from the visual records of proximity and distance cameras as well as the debrief mode records of the real-time devices.<br><br>**Assessment:** Posttest was conducted by two measurement researchers at right after the training and 4 weeks later to identify the continuity of the training effects.	**Control Group:** none. Resusci Anne manikin<br><br>**Experimental Group:** Resusci Anne	Resusci Anne	0 days (post-intervention)<br>4 weeks	**General:** Chest compression accuracy (calculated from compression depth, rate, release, and hand position), Mouth-to-mouth ventilation accuracy (assessed with the mouth-to-mouth ventilation scores), Cardiopulmonary resuscitation performance ability (measured using the 20-item CPR performance ability scale based on the 2015 Korean guideline for CPR).
Wagner et al [7]	2018	Austria	Randomized Simulation Study	653	Third-year medical students	**Previous**: Third-year medical students from the Medical University of Vienna who were required to do their mandatory pediatric CPR training were included.<br><br>**Upon study:** Before the training, participants reviewed the current pediatric CPR guidelines and watched a demonstration of the CPR algorithm by an instructor.	**2 manikin groups:**<br>**Group 1:** performed CPR training using a quality of cardiopulmonary resuscitation (QCPR) infant manikin<br>**Group 2:** performed QCPR training using a QCPR Resusci Anne (LaerdalMedical GmbH)<br><br>**3 feedback groups:**<br>**(1) instructor feedback (IF) group:** participants completed the CPR training with feedback from their instructor without the assistance of a feedback device<br>**(2) device feedback (DF) group**: participants received direct visual feedback from a feedback device during CPR training but did not received any feedback from an instructor;<br>**(3) instructor and device feedback (IDF) group**: participants received direct verbal feedback from an instructor who continuously observed the participants' actual CC quality on the feedback device.<br><br>**Training**: In the training phase, participants practiced CPR with feedback for 2 minutes.<br><br>**Assessment:** 2-minute CPR on the same manikin after 45 minute rest.	**Group 1:** Resusci Baby QCPR (Laerdal Medical GmbH, Stavanger, Norway) the SimPad touchscreen with the SkillReporter software (Laerdal);<br>**Group 2:** QCPR Resusci Anne (LaerdalMedical GmbH) the SimPad touchscreen with the SkillReporter software (Laerdal)<br><br>**DF group**: SimPad visible to the participant<br>**IDF group**: SimPad visible to the instructor only<br>**IF group**: SimPad masked to both	**Group 1:** Resusci Baby QCPR (Laerdal Medical GmbH, Stavanger, Norway) the SimPad touchscreen with the SkillReporter software (Laerdal) for data collection<br><br>**Group 2:** QCPR Resusci Anne (LaerdalMedical GmbH) the SimPad touchscreen with the SkillReporter software (Laerdal) for data collection	0 days (post-intervention)	**Primary:** total compression score<br><br>**Secondary:** all subcomponents of the total compression score, including correct hand position, mean CC depth, CC depth compliance, mean CC rate, CC rate compliance, and the proportion of complete release.
Smereka et al [8]	2019	Poland	Randomized Simulation Trial	94	First year nursing students	**Previous**: no previous participation in a CPR training.<br><br>**Uppon study**: Prior to the survey, all participants attended a standard training course in basic life support (BLS) based on the American Heart Association (AHA) 2015 guidelines, conducted by accredited AHA instructors. After a successful completion of the theoretical training, the participants underwent a 10-minute practical training during which they performed unassisted chest compressions.	**Training:** theoretical and 10-minute practical training during which performed unassisted chest compressions.<br><br>**Control group:** next day, practiced chest compressions without the use of any device for half an hour<br><br>**Experimental group:** next day, practiced with the use of TrueCPR for half an hour.<br><br>**Assessment:** Further measurement of chest compressions was performed after a month, when the participants were asked to perform a 2-minute unassisted (instrument-free) resuscitation cycle.	**Control group:** none<br><br>**Experimental Group:** TrueCPR on unknown manikin	unknown makinin	1 month	**General:** Frequency and depth of chest compressions, and the degree of full chest relaxation.<br><br>**Others:** self-assessed confidence of chest compression quality of the subjects was measured with a 100-point scale
Sá-Couto et al [9]	2018	Portugal	Randomized Control Study	39	Pregraduated students of Medicine and Biomedical Engineering	**Previous**: The recruitment of medical students was restricted to basic years in order to avoid students with CPR training.<br><br>**Upon study**: On Day 1, students also received the 2015 CPR guidelines1 and algorithm and were advised to review them.	**Control group:** trained with a standard torso and received instructions and feedback from an experienced Basic Life Support (BLS) instructor. The training time was equivalent for both groups and lasted approximately 1 hour for each subgroup of students.<br><br>**Intervention group:** trained with the prototype, receiving feedback on CPR relevant parameters: hands position, rate, and recoil, from the CPR Personal Trainer interface (without instructor). The training time was equivalent for both groups and lasted approximately 1 hour for each subgroup of students.<br><br>**Assessment:** Theoretical tests. Practical consisting in executing 2 minutes of the BLS algorithm. All performances were video recorded. The evaluation of the correct application of the BLS algorithm and correct CPR performance of each student was made through visualization of each video by an independent expert, blinded to the study, with the use of a checklist.	**Control Group:** none. Adult Brad (Simulaids) manikin<br><br>**Intervention Group:** CPR Personal Trainer on Adult Brad (Simulaids)	Video analysis and checklist	1 week	**Checklist:** call for help, check normal breathing, compressions-to-ventilations ratio of 30:2, compression rate, hands position, chest recoil, compressions depth, 2 deep breaths, pause for breaths of <10 seconds, and head extension.<br>Each item was rated as 0-incorrect/not applied, 1—insufficient/incomplete, and 2—correct.<br>The total score of the practical test was calculated as the mean of the 10 items with a maximum value of 2.<br><br>**Others:** SUS questionnaire
González-Santano et al [10]	2020	Spain	Randomized Trial	30	Beach Lifeguards	**Previous**: All lifeguards had been trained in BLS in the previous 2 years.<br><br>**Upon study**: 12-min session of training and practised at least 6 min of CPR.	**Traditional training (TT):** training with a manikin without feedback system guided by the instructor;<br>**App training (AP):** training using an app (Massage cardiaque et DSA)<br>**Feedback training (FT)**: training with a manikin with feedback<br><br>**Training:** The lifeguards received the same training; a 12-min session of training and practised at least 6 min of CPR.<br><br>**Assessment:** Between 7 and 15 days after the training, each lifeguard performed an evaluation of a 3-min CPR simulation scenario.	**Traditional training (TT)**: Resusci Anne manikin without feedback system<br><br>**App training (AP):** Massage cardiaque et DAS app<br><br>**Feedback training (FT):** Resusci Anne QCPR Skillreporter	Resusci Anne QCPR Skillreporter (Laerdal, Norway) for data collection	Between 7 and 15 days	**General:** Total number of compressions, % hands positioning, mm of depth achieved, % compressions at correct depth, % correct chest recoil, mean rate, % correct rate compressions, total number of ventilations, mean volume, % correct ventilations, and QCPR, QCC (quality of compressions = [(% compressions at correct depth + % correct chest recoil + % correct rate compressions)/3].)
Pavo et al [11]	2016	Austria	Randomised simulation study	326	Undergraduate third year medical students	**Previous**: Students with basic BLS skills were recruited from the second unit of a compulsory BLS training course of the curriculum.<br><br>**Upon study**: Upon inclusion in the study, all students had received 2 h of basic BLS training on a manikin following a standardised teaching protocol of the Medical University of Vienna according to the ILCOR guidelines for adult automated external defibrillator BLS (AED-BLS). Prior to study measurements, all participants received initial training using a modified 4-stage approach: following a standardised video according to their group, students were allowed to practice their basic life support (BLS) scenario under supervision until they felt sufficiently confident with the method. The duration of the training videos and the free practice differed between the different groups.	**Training:** standardised video according to each group, students were allowed to practice their basic life support (BLS) scenario under supervision until they felt sufficiently<br>confident with the method. The duration of the training videos and the free practice differed between the different groups.<br><br>**Standard BLS (sCPR) group:** performed resuscitation with a compression to ventilation ratio of 30:2 with a change of rescuer position after 2 min as recommended by the ERC 2010 guidelines<br><br>**human feedback (hfCPR) group:** The study participant performing ventilation was instructed to give verbal feedback about the aforementioned CPR parameters to the rescuer performing chest compressions at the beginning of each new cycle and to correct in any case of deviation from the trained criteria.<br><br>**QCPR feedback device (mfCPR) group:** performed BLS using the HeartStart MRx with Q-CPR-TechnologyTM (Philips, Netherlands) feedback device. Real time visual feedback is provided using graphs and numbers on the display of the MRxTM defibrillator placed next to the manikin. Furthermore, automated audio feedback advises the rescuer about necessary corrections if values diverge from the programmed range.<br><br>**Assessment:** After completion of training, participants performed 8 min of two-rescuer BLS with bag-valve mask ventilation according to ERC 2015 guidelines.	**standard BLS (sCPR) group:** none<br><br>**human feedback (hfCPR) group:** none<br><br>**QCPR feedback device (mfCPR) group:** HeartStart MRx with Q-CPR-TechnologyTM (Philips, Netherlands)	Ambu®ManC manikins (Ambu, Ballerup, Denmark) with the Ambu® CPR Software (version 2.3.9, Ambu®, Ballerup, Denmark) for data collection	0 days (post-intervention)	**Primary:** Effective compression ratio (ECR), a parameter combining correct hand position, chest compression depth, and complete decompression multiplied by flow-time fraction -> effective compressions in % (EC: correct hand position, depth (50–60 mm) and complete decompression) multiplied by flow-time fraction (FTF) in %.<br><br>**Secondary**: EC, compression rate (CR), compression depth, complete decompressions and incorrect pressure point were assessed. Additionally, time related parameters such as FTF (flow time fraction of chest compressions), absolute hands-off time (time fraction without compressions or ventilation) and time until first chest compression as well as ventilation parameters such as ventilation volume, ventilation minute volume, ventilation time and the number of gastric inflations<br><br>Subjective assessment by study participants
Baldi et al [12]	2017	Italy	Randomized controlled manikin study	450	Laypersons over 18 years old with no previous training in CPR	**Previous**: Laypersons over 18 years old with no previous training in CPR were eligible for randomization.<br><br>**Upon study**: Each group attended one of three different types of 5-hour BLS/AED courses. Every course consisted of 1 hour of theory and 4 hours of practice. The only difference among the courses was the amount of time spent training with feedback.	**Training:** Each group attended one of three different types of 5-hour BLS/AED courses. Every course consisted of 1 hour of theory and 4 hours of practice. The only difference among the courses was the amount of time spent training with feedback.<br><br>**Course NF:** simple BLS/AED course without any feedback<br>**Course SF**: BLS/AED course with 1 minute of training with real-time visual feedback manikin<br>**Course LF:** BLS/AED course with 10 minutes of training with real-time visual feedback manikin<br><br>**Assessment:** At the end of each course, we recorded 1 minute of compression-only CPR using the same software and the same manikin, without visual feedback for the attendee.	**Course NF:** none. Laerdal Little Anne manikin.<br><br>**Couse SF & LF:** Laerdal Little Anne (Laerdal Medical, Inc., AS, Norway) with Resusci Anne Wireless SkillReporter software, version 1.1.0.20 (Laerdal Medical, Inc., AS, Norway)	Laerdal Little Anne (Laerdal Medical, Inc., AS, Norway) with Resusci Anne Wireless SkillReporter software, version 1.1.0.20 (Laerdal Medical, Inc., AS, Norway) for data collection	0 days (post-intervention)	**General:** Percentage of compressions with correct depth (50 mm–60 mm), the percentage of correctly released compressions, the percentage of compressions with the correct hand position, the number of compressions, and the Total CPR Score<br><br>**Primary:** difference in the percentage of compressions with correct depth (50 mm–60 mm) among the groups.<br><br>**Secondary:** differences in the percentage of correctly released compressions, in the percentage of compressions with correct hand position, in the compression rate, and in the Total CPR Score among the groups.

Sarac [13]	2017	**Turkey**		76	Second-year university students of different academic disciplines enrolled in "First Aid " elective course	**Previous**: All were enrolled in "First Aid (FA)," an elective course provided by the School of Physical Education and Sports. One of the students was excluded from the study for having previous training in FA and CPR.<br><br>**Upon entry the study:** The FA course was a one semester (14 weeks) instructor-led course involving two hours of class time per week. Although the course covered topics, such as circulatory emergencies, respiratory emergencies, soft tissue injuries, and burns, this study only included the topic of CPR. The CPR section of the FA course lasted for two weeks (two hours a week).	**Training**: The FA course was a one semester (14 weeks) instructor-led course involving two hours of class time per week. Each group had four hours (two hours a week) of theoretical training and hands-on practice on a Resusci Anne Basic Torso CPR Manikin<br>During hands-on practice, students were observed and were provided feedback by the instructor when necessary.<br><br>**Control group:** no feedback<br>**AC (augmented concurrent) group:** realtime feedback from the device. Before administering the post-test, each student in the AC group did ten sets of CPR trials on a Laerdal Resusci Anne Manikin by using its real-time visual feedback features. At the end of this five-minute recovery period, students in the AC group were required to perform ten sets of CPR as a post-test.<br>**AT-KR (augmented terminal knowledge of results) group:** printed report of their CPR skills after the training. Students in AT-KR group were also given ten sets of CPR trial and a five-minute recovery period within the post-test. However, the real-time visual feedback features were not provided to students in the AT-KR group. They were given a printed report of their CPR ventilation and compression skill performance during the ten sets of CPR trial.<br><br>**Assessment:** Four weeks after the pre-test, all students in the three groups were required to perform ten sets of CPR for a post-test, which was administered in the same manner.	**Control group:** No information<br><br>**AC & ATKR Groups:** Resusci Anne Basic Torso CPR Manikin	Resusci Anne Basic Torso CPR Manikin with Laerdal Resusci Anne SkillReporter for data collection	4 weeks	**Ventilation skills:** average ventilation volume, average number of ventilations per minute, minute ventilation volume, total number of ventilations, number of correct ventilations, percent of correct ventilations, number of too many ventilations, number of too few ventilations, and number of too fast ventilations<br><br>**Compression skills:** average compression depth, average number of compressions per minute, average compression rate, total number of compressions, number of correct compressions, percent of correct compressions, number of too deep compressions, number of too shallow compressions, number of wrong hand positions, number of too low hand positions and number of incomplete releases
Jang et al [14]	2020	**South Korea**	Randomized simulation study	**95**	General university students	**Previous**: The inclusion criteria were age 19 years and university students. There were no exclusion criteria.<br><br>**Upon study**: All participants attended basic life support (BLS) and AED training program. The official BLS and AED training programs from the Korean Association of Cardiopulmonary Resuscitation were employed to train both groups for 1 hour each. The CPR course comprised theory and practice classes that included performing a 2-minute COCPR, four times.	**Training:** The official BLS and AED training programs from the Korean Association of Cardiopulmonary Resuscitation were employed to train both groups for 1 hour each. The CPR course comprised theory and practice classes that included performing a 2-minute COCPR, four times.<br><br>**Real-time feed-back group (RTFG):** training with feedback<br><br>**Non-feedback group (NFG):** training without feedback<br><br>**Assessment:** 2-minute COCPR skill immediately after the training. To evaluate skill retention, the groups were re-evaluated 3 , 6 , and 9 months after the training. No further feedback was provided after initial training.	**NF group:** no information<br><br>**RTFG group:** CPR training manikin (BT-SEEM; BT Inc., Goyang, Korea)	BT-CPTA (BT Inc.).	0 days (post intervention)<br>3 months<br>6 months<br>9 months	**General:** chest compression rate, the average depth of chest compression, the percentage of adequate chest compression depth, correct hand position (%), and chest recoil (%)
Zhou et al [15]	2020	China	Randomized controlled study	97	Third-year medical students	**Previous**: The characteristics of participants, including age, sex, height, weight, number of previous CPR trainings, and the experience of actual CPR, were recorded.<br><br>**Upon study**: 45-minute CC-only CPR training program that included the following: (1) instruction and practice ofcardiac arrest recognition and calling 1-2 0 (emergency call in China) for help, (2) instruction and practice of CC, and (3) use of an automated external defibrillator (AED). During training, instructors were asked to ensure each participant perform 5 cycles (2 min/cycle) of CC on manikins.	**Training:** 45-minute CC-only CPR training program. In each session, participants first practiced CC under the supervision of instructors for a total of 30 min.<br><br>**RP group:** performed repetitive sessions of CC-only CPR under the guidance of the instructor<br><br>**RP + AVF group:** performed repetitive sessions of CC-only CPR under the guidance of them instructor and the real-time AVF devices<br><br>**Assessment:** One hour after each session each participant performed a 2-min CC, and the quality of compressions was determined (short- time retention). long-time CC quality retention were performed at 3 months and 12 months after training.	**Both:** Laerdal Resusci Anne QCPR torso manikin (Laerdal China Ltd., Hangzhou, China) with SimPad Plus	Laerdal Resusci Anne QCPR torso manikin (Laerdal China Ltd., Hangzhou, China) with SimPad Plus for data collection	day 1 (post-intervention 1)<br>day 3 (post-intervention 2)<br>day 7 (post-intervention 3)<br>3 months<br>12 months	**General:** CC rate, depth, correct hand position, and complete chest recoil
Eshel et al [16]	2019	**Israel**	Randomized controlled study	145	First-year medical students	**Previous**: The study included 201 first-year medical students, from 2 consecutive classes, participating in a semestrial mandatory "First Aid and Emergency Medicine" course. Students with advanced medical experience and training were exempt from the<br>course and were not included in the study groups.<br><br>**Upon study:** The CPR module curriculum, based on the 2015 AHA guidelines, included a 1-hour lecture and 10 academic hours of review and hands-on CPR training in small groups, as well as additional none-mandatory training time before the final examination. The final examination of the course, performed at the end of academic semester, included a written segment and a standardized OSCE.	**Training**: 1-hour lecture and 10 academic hours of review and hands-on CPR training in small groups, as well as additional none-mandatory training time before the final examination. The only major difference between the groups was the use of real-time audiovisual feedback teaching aids in the intervention group.<br><br>**Control group:** taught CPR on manikins without feedback capabilities<br><br>**Intervention group:** CPR training with an added real-time feedback audiovisual device<br><br>**Assessment:** No information	**Control Group**: Laerdal Resusci Anne<br><br>**Intervention group**: Laerdal Resusci Anne QCPR manikin accompanied by the handheld Simpad Skillreporter apparatus (Laerdal Medical AS, Stavanger, Norway).	Laerdal Resusci Anne QCPR manikin accompanied by the handheld Simpad Skillreporter apparatus (Laerdal Medical AS, Stavanger, Norway) for data collection	**post intervention**	**Primary:** Total CPR Score, Chest Compression Fraction (CCF), Compressions with Adequate Depth (percentage of compressions with a measured depth of ≥50 mm)<br><br>**Secondary:** "Other inclusive parameters calculated by the manikin"
Katipoglu et al [17]	2021	**Poland**	Randomized simulation study	111	First year students of medicine	**Previous:** unknown**.**<br><br>**Upon study:** In both groups a 5 minutes standardized training on how to perform CPR of an adult was performed prior to the study. Both groups then underwent a basic life support (BLS) course based on the AHA guidelines, with the first group (experimental group) performed chest compressions to observe, in real-time, chest compressions parameters indicated by software included in the simulator, and the second group (control group) performed compressions without the possibility of observing simulator indications. After a 10-minute resuscitation, the participants had a 30-minute break and then a 2-minute cycle of CPR based on a scheme of 30 compressions: 2 rescue breaths. The first group performed compressions on the basis of simulator indications, while the second group did not.	**Training:** In both groups a 5 minutes standardized training on how to perform CPR of an adult was performed prior to the study and a 10-minute resuscitation.<br><br>**Experimental group:** performed chest compressions to observe, in real-time, chest compressions parameters indicated by software included in the simulator.<br><br>**Control group:** performed compressions without the possibility of observing simulator indications.<br><br>**Assessment:** The participants had a 30-minute break and then a 2-minute cycle of CPR. Experimental group was able to observe real-time measurements Repeated assessment 1 month after training. Both experimental and control groups were not able to observe real-time measurements regarding quality of chest compression.	**Control group:** no information<br><br>**Experimental group**: Resusci Anne® QCPR (Laerdal, Stavanger, Norway)	Resusci Anne® QCPR (Laerdal, Stavanger, Norway) for data collection	**post intervention**<br><br>1 month	**General:** total compression score, compression depth, compression depth compliance, compression rate per minute (CPM), compression rate compliance, full release, correctness of chest position
Tanaka et al [18]	2019	Japan	Cluster randomized control trial	497	CPR layperson trainees	**Previous**: . The exclusion criteria were working as a healthcare professional regularly involved in resuscitation, such as Emergency Medical Technicians, paramedics and emergency room physicians or nurses. Previous CPR training status and quantity and timing of previous trainings were not used as inclusion or exclusion criteria.<br><br>**Upon study**: The training started with a PowerPoint presentation-based instructor-led lecture followed by psychomotor practice. Psychomotor practice focused on chest-compression CPR.	**Control group:** standard CPR training with instructor's subjective feedback based on the instructor's experience<br><br>**QCPR Classroom group:** subjective and objective feedback from the instructor based on real-time feedback through the manikin, and were able to correct themselves based on feedback displayed on the screen of the device.<br><br>**Assessment:** 1min of chest compression was measured after the training as a post-test.	**QCPR group**: Laerdal QCPR Classroom manikin system (Laerdal Medical, Stavanger, Norway)<br><br>**Control group**: Laerdal Little Anne manikins. However, we did not use a<br>screen to show students objective feedback.	Laerdal QCPR Classroom manikin system (Laerdal Medical, Stavanger, Norway) for data collection	0 days (post intervention)	**Primary:** compression depth (mm), compression rate (cpm), adequate depth (%) and adequate recoil (%).<br><br>**Secondary:** scores from a survey conducted after the training

Labuschagne et al [19]	2022	South Africa	**Experimental Study**	53	Final-year (fifth year of study) undergraduate medical students	**Previous**: all participants in the study had previously received the same CPR training (via the con- ventional CPR training method) in their third year of the medical under- graduate programme.<br><br>**Upon study:** In their fifth year, students receive CPR training in the Anaesthesiology rotation and in the Family Medicine rotation. The fifth-year undergraduate medical students rotating at Anaesthesi- ology attended scheduled BLS lectures as part of their Anaesthesiology curriculum. A different group of fifth-year students (consisting of 6–8 students) attended the lecture every two weeks. This study was con- ducted during the Anaesthesiology CPR training session	**Training:** All the participants received the same one-hour theoretical lecture on CPR, presented by the same anaesthesiologist with more than 30 years' experience as an educator. Thereafter, the participants were divided into two groups for practical training.<br><br>**Control Group (CPR)**: The CPR group, in a different room, were given the opportunity to practice using conventional CPR manikins.Students in both groups were allowed to practice (in separate rooms) for approximately 10 minutes.<br><br>**QCPR Group:** The QCPR group was put into a room and given access to the QCPR manikins and tablets with the programme. Students in both groups were allowed to practice (in separate rooms) for approximately 10 minutes.<br><br>**Assessment**: After the 10-minute practical training session, both groups were tested using the QCPR manikin and programme**.** The practical test was done for two minutes per person as this is one typical cycle in practical CPR before the medical practitioner switches with another to avoid exhaustion and to maintain effectiveness.	**Control Group (CPR):** Conventional CPR manikins<br><br>**QCPR Group:** electronic-feedback QCPR manikins	QCPR manikins	Post intervention	**General**: Flow fraction, Average compression rate per minute, Compressions of adequate depth, Compressions of adequate rate, Total effectiveness percentages
Lee et al [20]	2023	Taiwan	Randomised Controlled Study	90	Nurses	**Previous**: A recertification program for the CG participants was arranged in the same week, whereas that for the EG participants was conducted in the following week.<br><br>**Upon study**: Participants received a 10-min standardized lecture that provided up-to-date information on high-quality CPR. Subsequently, all participants practiced CPR skills for 30 min in a kneeling position on a Little Anne QCPR manikin.	**Training**: Participants received a 10-min standardized lecture that provided up-to-date information on high-quality CPR. Subsequently, all participants practiced CPR skills for 30 min in a kneeling position on a Little Anne QCPR manikin.<br><br>**Control**: Participants in the CG were judged on the basis of instructors' visual perception; instructors advised participants for improving their skills.<br><br>**Intervention**: Participants in the EG adjusted their skills according to the real-time visual feedback data displayed on an iPad in front of them.<br><br>**Assessment**: The postintervention test, consisting of 5 cycles of CPR and the selfefficacy questionnaire, was administered to both groups immediately after the recertification program (T1) and 12 weeks later (T2).	**Control group:** Little Anne QCPR, Laerdal Medical<br><br>**Experimental group**: Little Anne QCPR, Laerdal Medical	Little Anne QCPR, Laerdal Medical	Post intervention<br>12 months	**Primary:** group differences noted in performance parameters after 5 cycles of CPR: (a) appropriate rate: percentage of chest compression counts between 100 and 120 per min, (b) appropriate depth: percentage of chest compression depth between 5 and 6 cm, (c) appropriate chest recoil: percentage of fully recoiled chest after each compression, (d) chest compression factor (CCF): percentage of time spent on providing chest compressions, and (e) the total score of chest compression quality: a summed score of appropriate rate, depth, and chest recoil.<br><br>**Secondary:** group differences noted in self-efficacy measured using a questionnaire developed by modifying the Basic Resuscitation Skills Self-Efficacy Scale

REFERENCES

1 Cortegiani A, Russotto V, Montalto F, Iozzo P, Meschis R, Pugliesi M, et al. Use of a real-time training software (Laerdal QCPR®) compared to instructor-based feedback for high-quality chest compressions acquisition in secondary school students: a randomized trial. PLoS One. 2017;12(1):e0169591.
2 Lin Y, Cheng A, Grant VJ, Currie GR, Hecker KG. Improving CPR quality with distributed practice and real-time feedback in pediatric healthcare providers—a randomized controlled trial. Resuscitation. 2018;130:6-12.
3 Chamdawala H, Meltzer JA, Shankar V, Elachi D, Jarzynka SM, Nixon AF. Cardiopulmonary resuscitation skill training and retention in teens (CPR START): a randomized control trial in high school students. Resusc Plus. 2021;5:100079.
4 Moreno S, Sisó-Almirall A, Kostov B, Expósito M, Moreno JR, de Pablo B, et al. Cardiopulmonary resuscitation skill maintenance for primary care staff: brief training sessions with feedback. Emergencias. 2021;33(3):203-210.
5 Suet G, Blanie A, de Montblanc J, Roulleau P, Benhamou D. External cardiac massage training of medical students: a randomized comparison of two feedback methods to standard training. J Emerg Med. 2020;59(2):270-277.
6 Kim Y, Han H, Lee S, Lee J. Effects of the non-contact cardiopulmonary resuscitation training using smart technology. Eur J Cardiovasc Nurs. 2021;20(8):760-766.
7 Wagner M, Bibl K, Hrdliczka E, Steinbauer P, Stiller M, Gröpel P, et al. Effects of feedback on chest compression quality: a randomized simulation study. Pediatrics. 2019;143(2):e20182441.
8 Smereka J, Szarpak L, Czekajlo M, Abelson A, Zwolinski P, Plusa T, et al. The TrueCPR device in the process of teaching cardiopulmonary resuscitation: a randomized simulation trial. Medicine (Baltimore). 2019;98(27):e15995.
9 Sá-Couto C, Ferreira AM, Almeida D, Nicolau A, Vieira-Marques P. Evaluation of skills acquisition using a new low-cost tool for CPR self-training. Porto Biomed J. 2018;3(1):e8.
10 González-Santano D, Fernández-García D, Silvestre-Medina E, Remuiñán-Rodríguez B, Rosell-Ortiz F, Gómez-Salgado J, et al. Evaluation of three methods for CPR training to lifeguards: a randomised trial using traditional procedures and new technologies. Medicina (Kaunas). 2020;56(11):577.
11 Pavo N, Goliasch G, Nierscher FJ, Stumpf D, Haugk M, Breckwoldt J, et al. Short structured feedback training is equivalent to a mechanical feedback device in two-rescuer BLS: a randomised simulation study. Scand J Trauma Resusc Emerg Med. 2016;24:70.
12 Baldi E, Cornara S, Contri E, Epis F, Fina D, Zelaschi B, et al. Real-time visual feedback during training improves laypersons' CPR quality: a randomized controlled manikin study. CJEM. 2017;19(6):480-487.
13 Sarac L. Effects of augmented feedback on cardiopulmonary resuscitation skill acquisition: concurrent versus terminal. Eurasian J Educ Res. 2017;17(72):83.
14 Jang TC, Ryoo HW, Moon S, Ahn JY, Lee DE, Lee WK, et al. Long-term benefits of chest compression-only cardiopulmonary resuscitation training using real-time visual feedback manikins: a randomized simulation study. Clin Exp Emerg Med. 2020;7(3):206-212.
15 Zhou XL, Wang J, Jin XQ, Zhao Y, Liu RL, Jiang C. Quality retention of chest compression after repetitive practices with or without feedback devices: a randomized manikin study. Am J Emerg Med. 2020;38(1):73-78.
16 Eshel R, Wacht O, Schwartz D. Real-time audiovisual feedback training improves cardiopulmonary resuscitation performance: a controlled study. Simul Healthc. 2019;14(6):359-365.
17 Katipoglu B, Madziala MA, Evrin T, Gawlowski P, Szarpak A, Dabrowska A, et al. How should we teach cardiopulmonary resuscitation? Randomized multi-center study. Cardiol J. 2021;28(3):439-445.
18 Tanaka S, Tsukigase K, Hara T, Sagisaka R, Myklebust H, Birkenes TS, et al. Effect of real-time visual feedback device 'Quality Cardiopulmonary Resuscitation (QCPR) Classroom' with a metronome sound on layperson CPR training in Japan: a cluster randomized control trial. BMJ Open. 2019;9(6):e026140.
19 Labuschagne MJ, Arbee A, de Klerk C, de Vries E, de Waal T, Jhetam T, et al. A comparison of the effectiveness of QCPR and conventional CPR training in final-year medical students at a South African university. Afr J Emerg Med. 2022;12(2):106-111.
20 Lee PH, Lai HY, Hsieh TC, Wu WR. Using real-time device-based visual feedback in CPR recertification programs: a prospective randomised controlled study. Nurse Educ Today. 2023;124:105755.
