# Supplementary figures and images for: Influence of Training With Corrective Feedback Devices on Cardiopulmonary Resuscitation Skills Acquisition and Retention: Systematic Review and Meta-Analysis
Source: JMIR Med Educ. 2024 Dec 19;10:e59720. doi: 10.2196/59720 (PMC11695954; doi:10.2196/59720)

Multimedia Appendix 4: Publication bias—funnel plot.

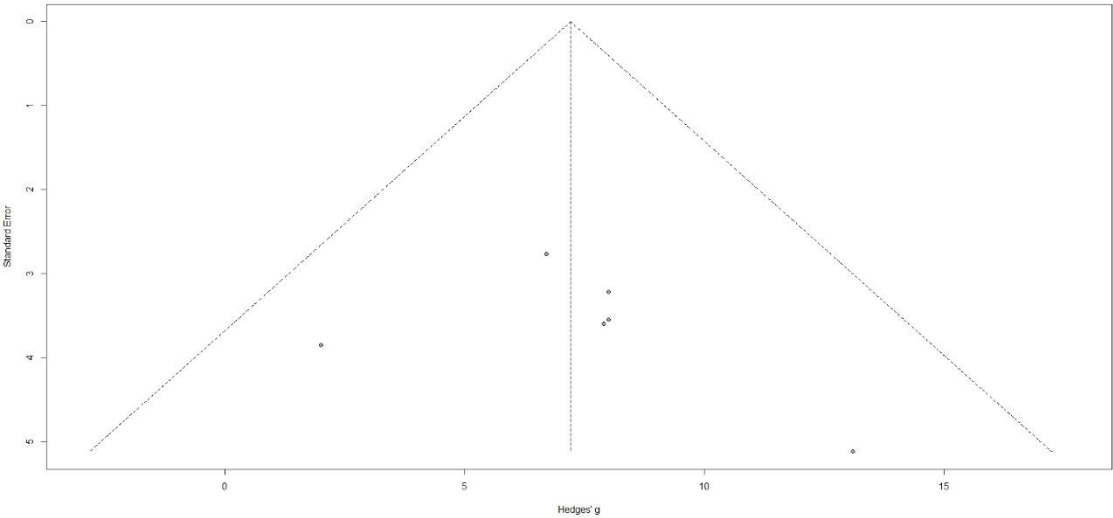

Supplement: Multimedia Appendix 4 [file mededu_v10i1e59720_app4.pdf]
